# Mesenchymal stromal cells attenuate post-stroke infection by preventing caspase-1-dependent splenic marginal zone B cell death

**DOI:** 10.1038/s41392-020-00415-0

**Published:** 2021-02-12

**Authors:** Yinong Huang, Jiancheng Wang, Xiaofan Lai, Yuan Qiu, Jianye Cai, Yuanchen Ma, Yilin Liu, Wei Qiu, Zhengqi Lu, Andy Peng Xiang

**Affiliations:** 1 Department of Neurology, The Third Affiliated Hospital, Sun Yat-Sen University, Guangzhou, China; 2 Center for Stem Cell Biology and Tissue Engineering, Key Laboratory for Stem Cells and Tissue Engineering, Ministry of Education, Sun Yat-Sen University, Guangzhou, China; 3 The Seventh Affiliated Hospital, Sun Yat-sen University, Shenzhen, China

**Keywords:** Mesenchymal stem cells, Diseases of the nervous system

**Dear Editor**,

Spontaneous infection is one of the most common complications of acute ischemic stroke (AIS) and leads to increased morbidity and mortality in stroke patients. However, current interventions fail to improve clinical outcomes in these patients. Since stroke-induced immunodeficiency is thought to contribute to the risk of infection,^1^ we hypothesized that mesenchymal stromal cell (MSC) therapy, which harnesses potent immunomodulatory capacities, could be a potential solution. Here, we show that intravenously administered MSCs preferentially migrate to the marginal zone (MZ) of the spleen, preserve the injured MZ B cells and ameliorate post-stroke infection and mortality in the mouse middle cerebral artery occlusion (MCAO) model of AIS.

To address the therapeutic effects of MSCs in post-stroke infections, male C57BL/6 mice were intravenously treated with MSCs at 6 h after reperfusion. To rule out the general benefits of cell therapy, we applied the treatment of human dermal fibroblasts (hDF) as control. Impressively, MSC-treated mice exhibited higher survival rate and smaller infarct volume compared to PBS-treated group (Fig. 1a and Supplementary Fig. 1). Furthermore, MSC therapy significantly reduced bacterial loads and rescued spleen shrinkage at 5 days after MCAO (Fig. 1b–d). Based on the protective effects of MSCs in the spleen, we examined the bio-distribution of MSCs after transplantation through transducing the cells with lentiviral vector encoding tdTomato. The infused MSCs were transiently observed in the lung but rapidly disappeared from this region after 1 day, while numerous alive MSCs were detected in the spleen during the first 5 days after transplantation (Supplementary Fig. 2a–c). More specifically, the transplanted MSCs mainly distributed in the MZ of the white pulp (Fig. 1e). The MZ is a highly transited area that facilitates filtration of blood-borne pathogens and initiates immune responses.^2^ The lymphocytes found in this area are principally MZ B cells, serving as the gatekeeper against infections.^2^ As expected, the infused MSCs were found near MZ B cells (Fig. 1f), indicating the possible interaction between MSCs and MZ B cells.

**Fig. 1:**
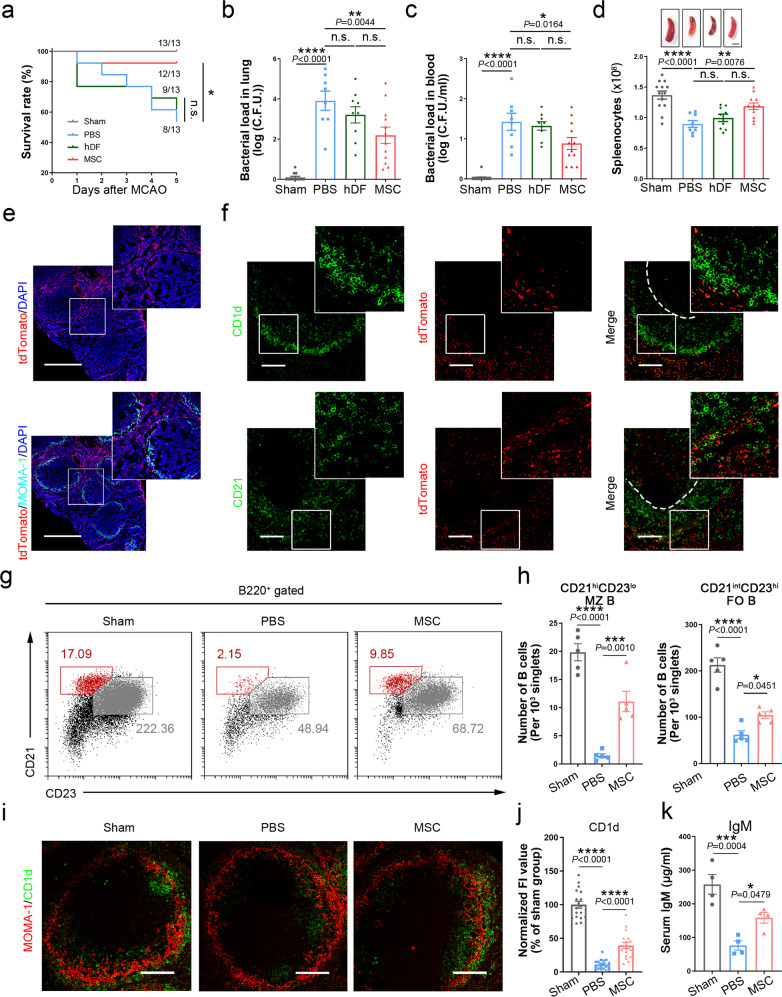
MSC administration alleviates post-stroke immunodepression and spontaneous infection by protecting MZ B cells. **a** The survival rate of each batch of mice was recorded daily for 5 days after MCAO. **b**, **c** Quantification of bacterial load in lung (**b**) and blood (**c**) samples of sham or post-stroke mice at 5 days post-MCAO. Data are presented as colony-forming units (CFU)/mL (log 10). **d** Quantification of the number of splenocytes in sham and MCAO mice treated with or without MSCs. Representative photographs of spleens from the different groups at 5 days after stroke. Scale bar: 2 mm. **e** Representative images showing that tdTomato-labeled MSCs mainly distributed in the marginal zone of the white pulp and red pulp at 3 days post-transplantation. Immunolabeling of MZ macrophages (light blue, MOMA-1) was used to identify the MZ. Scale bar: 500 μm. **f** Sections were obtained 3 days post-injection and stained with anti-CD1d or anti-CD21 (green) to label MZ B cells. tdTomato^+^ MSCs were observed in the vicinity of MZ B cells. Dashed lines outline the boundaries of the MZ B cell area. Scale bar: 100 μm. **g**, **h** Representative FACS panels (**g**) and quantification (**h**) analyses showed that MSC treatment induced a significant increase of MZ B cells and a less marked increase in FO B cells. *n* = 5. **i** Co-immunostaining for MOMA-1 (surface marker of MZ macrophages indicating the localization of MZ, red) and the MZ B cell markers, CD1d (green). Scale bar: 100 μm. **j** The mean fluorescence intensity (FI) of CD1d of MCAO mice were normalized with respect to the sham-operated group. *n* = 3 mice per group. Six independent fields of view per mouse. **k** Serum IgM levels were significantly higher in MSC-treated mice compared to PBS-treated controls. *n* = 4. FSC, forward scatter; SSC, side scatter; hi, high; int, intermediate; lo, low. Data are expressed as mean ± SEM and were assessed by the log-rank test (**a**) one-way ANOVA (**b**–**d**, **h**, **j**, **k**). *P < 0.05, **P < 0.01, ***P < 0.001

We further evaluated the immunomodulatory impacts of MSCs on splenocytes and observed striking reductions in T and B cells at 5 days post-MCAO, but MSC administration rescued this B cells loss (Supplementary Fig. 3). FACS analysis showed the number of B220^+^CD21^hi^CD23^lo^ MZ B cells and B220^+^CD21^int^CD23^hi^ follicular B cells were increased in the MSC group compared with the PBS group, with a greater difference seen among MZ B cells (Fig. 1g, h). Moreover, we demonstrated that MSC treatment effectively rescued the loss of immunoreactivity for these MZ B cell markers and increased circulating IgM levels (Fig. 1i, j). Given that MSCs in the MZ mainly distributed near MZ B cells and innate-like MZ B cells play a critical role in rapid anti-bacterial defense, we sought to determine the potential mechanisms of MZ B cells loss and how MSC administration preserved this subset of B cells.

Lymphocyte apoptosis is one of the main factors contributing to the post-stroke splenic atrophy.^1^ Here, a large percentage of CD1d^+^MZ B cells co-localized with TUNEL labeling in splenic sections from PBS-treated mice at 5 days post-MCAO, and MSC treatment decreased the population of CD1d^+^TUNEL^+^MZ B cells (Supplementary Fig. 4a, b). However, TUNEL labeling of fragmented DNA has been observed not only in classical apoptotic cells, but also in caspase-1-dependent pyroptosis.^3^ Therefore, we applied fluorescently labeled inhibitor of caspases (FLICA) probes to determine the distribution of active caspase-3 and caspase-1. Surprisingly, CD1d^+^MZ B cells were found to be co-localized with active caspase-1, but not active caspase-3. Meanwhile, a large fraction of the MZ B cells exhibited caspase-1 activation at 5 days post-MCAO, which was reduced by MSC treatment (Supplementary Fig. 4c–e). Furthermore, we revealed that experimental stroke-induced caspase-1 activation and GSDMD-mediated cell lysis in MZ B cells, and that MSC administration decreased this inflammatory form of programmed cell death (Supplementary Fig. 4f). A series of cytosolic receptors known as the NOD-like receptors (NLRs) have been found involved in inflammasome formation and mediate caspase-1 activation. So, we measured the mRNA levels of various NLRs in MZ B cells. In comparison to NLRP3, the expression levels of NLRP1 and NLRC4 were markedly lower. Additionally, MCC950 (an inhibitor of NLRP3) treatment reduced caspase-1 activation and subsequent GSDMD-mediated MZ B cells lysis (Supplementary Fig. 4g, h). These results indicate that the NLRP3-caspase-1 pathway plays an essential role in stroke-induced MZ B cell death and MSC infusion reduces caspase-1 activity in these MZ B cells, potentially through the inhibition of NLRP3 activation.

Increasing evidence suggests that damaged/dysfunctional mitochondria may release mitochondrial reactive oxidative species (mtROS), which could bind to and activate the NLRP3 inflammasome.^4^ Here, we exposed experimental animals to a mitochondria-targeted antioxidant (mito-TEMPLE) for 5 days after MCAO. The protein levels of NLRP3, cleaved caspase-1, and cleaved GSDMD were decreased in mice treated with mito-TEMPLE. As expected, the number of MZ B cells was also rescued by administration of mito-TEMPLE (Supplementary Fig. 5a, b). Besides, the excessive mtROS generation and disrupted mitochondrial membrane potential (MMP) in the MZ B cells after MCAO were alleviated by MSC treatment (Supplementary Fig. 5c–f). Considering the injected MSCs were distributed near MZ B cells, this might reflect the occurrence of direct mitochondrial transfer. To test this hypothesis, we labeled MSCs with mito-tracker prior to cell transplantation. Mitochondria from MSCs (mito-tracker^+^) were detected in CD1d^+^ MZ B cells at 5 days after MCAO, indicating that mitochondria were transferred from transplanted MSCs to injured MZ B cells (Supplementary Fig. 5g). Several previous studies have described the formation of microtubule for mitochondria transfer between MSCs and injured cells.^5^ Accordingly, we detected that mitochondria from MSCs were transferred via microtubule and fused with mito-tracker-red-labeled residual mitochondria in injured MZ B cells (Supplementary Fig. 5h, i). Taken together, these results illustrate that mitochondria from MSCs are transferred via microtubules to dying MZ B cells, where they fuse with dysfunctional mitochondria and rescue them from cell death.

In summary, our data demonstrated that the infused MSCs migrate to the splenic MZ, transfer functional mitochondria to dying MZ B cells, and protect these cells from NLRP3-caspase-1-dependent cell death, thereby preserving the capacity of spleen to mount a rapid defense against post-stroke infection.

## Supplementary information

Supplementary materials

## Data Availability

All data supporting this study are available from corresponding authors upon reasonable request.
