# Supplementary material for: Mesenchymal stromal cells attenuate post-stroke infection by preventing caspase-1-dependent splenic marginal zone B cell death
Source: Signal Transduct Target Ther. 2021 Feb 12;6:60. doi: 10.1038/s41392-020-00415-0 (PMC7881133; doi:10.1038/s41392-020-00415-0)
Supplement: Supplementary file 1 — Supplementary materials [file 41392_2020_415_MOESM1_ESM.pdf]

Mesenchymal stromal cells attenuate post-stroke infection by preventing caspase-1-dependent splenic marginal zone B cell death

This file includes:

Materials and Methods

References for “Materials and Methods” section

Supplementary Figures

Supplementary Figure 1. MSC administration reduced infarct size.

Supplementary Figure 2. Infused MSCs preferentially migrate to the spleen.

Supplementary Figure 3. MSC therapy prevents loss of splenic B cells.

Supplementary Figure 4. MSCs attenuate caspase1-dependent MZ B cell death, potentially through the inhibition of NLRP3 activation.

Supplementary Figure 5. MSCs transfer mitochondria to MZ B cells and maintain mitochondrial homeostasis in MZ B cells.

Materials and Methods

Animals and experimental stroke model

Male C57BL/6 mice aged 8 to 10 weeks were purchased from Guangdong Medical Laboratory Animal Center and housed in individually ventilated cages under specific-pathogen-free (SPF) conditions. A standard 12 h light/dark cycle was set, and the animals were given free access to food and water. All animal experimental procedures were approved by the Ethical Committee of Sun Yat-Sen University.
To generate the MCAO model, mice were anesthetized with 1.5% isoflurane in a 30% O_2/69% N_2O mixture and randomly assigned to the sham group or MCAO group. All surgical instruments were sterilized before operation. As previously described,[1] a 10-mm incision was made on the right side of neck and the carotid artery, external carotid artery (ECA), and internal carotid artery (ICA) were exposed. A silicone-coated suture was inserted into the ECA and then through the ICA to block the MCA. After 40 min, the suture was removed for the purpose of reperfusion, and the incision was carefully sutured. Mice of the sham group underwent the same isoflurane anesthesia and surgical procedures as the MCAO group, except there was no insertion of an intraluminal filament. The body temperature of all experimental mice was maintained at 37°C throughout the surgery and recovery using a heating pad. The inclusion criteria for tMCAO were: 1) regional cerebral blood flow decreases ＞70% during occlusion as detected by laser Doppler flowmetry; 2) neurological deficits 3 hour after MCAO with neurological score between 2~3 points (determined as described).[2] 6 hours later, the experimental mice were randomly assigned to PBS and MSC-treatment groups for further investigations.

Isolation, characterization, and transplantation of MSC/hDF

Heparin-treated bone marrow was obtained from healthy donors along with their informed consent. Each bone marrow aspirate was diluted 1:1 with human MSC culture medium (low-glucose DMEM with 10% fetal bovine serum, HyClone). MSCs were separated using density gradient centrifugation in Ficoll-Paque (1.077 g/mL, Amersham Biosciences) and seeded at $1x10^5$ cells/cm^2 in the MSC culture medium as passage 0. Nonadherent cells were removed by a complete change of the culture medium, and when the remaining cells reached 80%-90% confluence, they were continuously passaged using 0.25% trypsin-EDTA. For MSC administration, $1x10^6$ cells (passages 4-8) were suspended in 0.1 ml PBS and transplanted via the caudal vein at 6 h after MCAO. Besides, hDFs were isolated from the foreskin of adult healthy donor with informed consent and cultured as previously described.[3] These fibroblasts were adopted as the cell control in this study.

Bacteriological analysis

Mice were euthanized at the indicated time points after MCAO and washed with 75% ethanol. Blood samples were obtained via cardiac puncture, and lung tissues were mechanically separated, minced, and homogenized under sterile conditions. To measure bacteria in terms of colony-forming units (C.F.U.), 10 μl blood or lung homogenate was serially diluted with PBS, plated on blood agar plates, and incubated at 37°C. After 24 h, the bacterial colonies were counted by researchers blinded to the treatment assignment.

Flow cytometry

Experimental mice were euthanized at 1, 3, and 5 days after MCAO and perfused with ice-cold saline, and the spleen, lung, and ipsilateral hemisphere tissues were isolated. Single cells from these tissues were prepared for immunostaining and FACS analysis. Spleen samples were mechanically homogenized and filtered through 40-μm cell strainers. 1X Red blood cell (RBC) lysis buffer (BioGems) was used to remove RBCs from the splenic cell suspensions. Lung tissues were collected, dissected, homogenized, digested in 0.25% trypsin at 37°C for 30 min, and passed through 40-μm filters. For brains, the ipsilateral hemispheres were digested with 0.25% trypsin at 37°C for 30 min and pressed through 40-μm filters, and then the myelin debris was removed from brain cells using gradient centrifugation in 30%/70% Percoll solution (Sigma-Aldrich).

Splenic cell suspensions were stained with CD3e-PE (1:400, BD Bioscience), B220-FITC (1:400, BD Bioscience), CD21/CD35-APC (1:400, Biolegend), CD23-BV421 (1:400, BD Bioscience), and fluorescently labeled inhibitor of caspases (FLICA) probes (1:100, ImmunoChemistry Technologies). FACS was performed using CytoFLEX and CytoFLEX s (Beckman Coulter) flow cytometers and assessed with the CytExpert and FlowJo X 10.0.7r2 software packages.

Immunofluorescence

At 3 days after MCAO, mice were euthanized and perfused with ice-cold saline followed by 4% paraformaldehyde (PFA). Spleen samples were collected, fixed overnight in 4% PFA, and cut into 20-μm frozen cryosections. Both splenic sections and cell attached to glass slides were fixed in 4% PFA for 20 min, permeabilized in 0.15% Triton X-100 for 15 min, blocked with PBS-5% normal goat serum for 1 h, and finally incubated with the appropriate antibodies, which included CD3e-PE (1:50, BD Bioscience), B220-FITC (1:50, BD Bioscience), MOMA-1-FITC (1:50, Biolegend), MOMA-1-PE (1:50, Biolegend), CD1d-FITC (1:50, BD Bioscience), CD21/CD35-APC (1:50, Biolegend), FLICA probes (1:50, ImmunoChemistry Technologies), anti-human COX4 antibody (1:100, Santa Cruz). Nuclei were visualized by DAPI (Fluka) staining for 10 min. Immunofluorescent images were acquired using an LSM800 confocal microscope (Zeiss), an LSM880 confocal microscope (Zeiss), and Dragonfly high-

speed confocal microscopy (ANDOR, Oxford Instruments). The supplementary movie was edited using the Imaris microscopy image analysis software (Oxford Instruments).

Enzyme-linked immunosorbent assay (ELISA)

A commercially available ELISA kit for anti-mouse IgM (RayBiotech) was used as recommended by the manufacturer to determine the IgM levels in the sera of experimental mice.

Western blotting

$B220^{+}CD21^{hi}CD23^{lo}$ MZB cells were collected using FACS and lysed with 1X RIPA buffer, and whole-cell proteins were extracted. The protein concentration was determined by a BCA protein assay kit (Thermo Fisher Scientific). Protein samples were separated by SDS-PAGE and transferred to a 0.45-μm pore-sized polyvinylidenedifluoride (PVDF) membrane (Millipore). Each membrane was blocked with 5% BSA and incubated with the appropriate antibodies, which included anti-caspase-1 (1:500, Santa Cruz), anti-Gasdermin D (1:1000, Cell Signaling Technology), anti-NLRP3 (1:1000, R&D Systems), and anti-GAPDH (1:3000, Cell Signaling Technology).

Mitochondrial membrane potential and ROS assessment

Mitochondrial membrane potential was detected using the cell-permeant dye, tetramethylrhodamine (TMRM, Thermo Fisher Scientific), which accumulates in mitochondria with intact membranes. To assess the levels of mitochondrial ROS, MZ B cells were labeled with fluorescent probe, MitoSOX Red (Thermo Fisher Scientific). Cells were incubated with the indicated dyes at 37°C for 30 min and analyzed by flow cytometry.

RNA isolation, reverse transcription, and quantitative real-time polymerase chain reaction (qRT-PCR)

Total RNA was extracted from isolated MZ B cells using the RNeasy mini kit (QIAGEN), according to the manufacturer's instructions. RNA (1 μg per sample) was subjected to reverse transcription using a RevertAid First Strand cDNA Synthesis Kit (Thermo Fisher Scientific). qRT-PCR was performed in duplicate using the SYBR Green qRT-PCR Super Mix (Roche) and detected by a Light Cycler 480 Detection System (Roche) as previously described.[4] The primers used for qRT-PCR analysis were: (mouse) NLRP1 F, GGTGGTGTGAAGATGTTGTGT and NLRP1 R, TCCATGTTCATCGTAGGGACC; NLRP3 F, ATTACCCGCCCGAGAAAGG and NLRP3 R, CATGAGTGTGGCTAGATCCAAG; NLRC4 F, ATCGTCATCACCGTGTGGAG and NLRC4 R, GCCAGACTCGCCTTCAATCA; β-actin F, GGCTGTATTCCCCTCCATCG and β-actin R, CCAGTTGGTAACAATGCCATGT; (human) CO1 F, CGCCACACTCCACGGAAGCA and CO1 R,

CGGGGCATTCCGGATAGGCC; and CO2 F, TTCATGATCACGCCCTCATA and CO2 R, TAAAGGATGCGTAGGGATGG.

Drug administration

MCC950 (Millipore) was dissolved in sterile PBS and intraperitoneally (i.p.) administered (10 mg/kg/d) to mice for 5 consecutive days starting at 6 h post-reperfusion. Mito-TEMPO (Sigma-Aldrich) was given i.p. at the same time points as MCC950, at 1 mg/kg/d. Mice in the sham and vehicle groups received equal volumes of saline (i.p.) and served as control groups. All animals were sacrificed at 5 days after MCAO for further analysis.

Lentiviral vector transduction

pLV/puro-EF1a-tdTomato was used to trace injected MSCs, as previously mentioned.[2] The MITO-GFP plasmid used to label mitochondria in MSCs was designated as pCMV-TOM20-EGFP-Neo (EX-G0283-M03, GeneCopeia). Lentivirus particles were harvested from 293FT cells that had been transiently cotransfected using Lipofectamine 2000 (Invitrogen). Harvested lentivirus particles were filtered through a 0.45-μm cell strainer and tittered. MSCs (passages 3 or 4) were transduced with the indicated lentivirus and purified using a flow cytometer (Influx, Becton Dickinson), as previously described.[5]

Statistical analysis

All results are presented as the mean ± S.E.M. of at least three independent experiments. The sample size of each experiment is indicated in the corresponding figure legend. One-way analysis of variance (ANOVA) was used to compare means among multiple groups. Survival curves were compared using the log-rank test. A *P*-value less than 0.05 was considered statistically significant.[5]

References

1 Wong, C. H., Jenne, C. N., Lee, W. Y., Leger, C. & Kubes, P. Functional innervation of hepatic iNKT cells is immunosuppressive following stroke. *Science* **334**, 101-105 (2011).

2 Huang, Y. *et al.* Targeted homing of CCR2-overexpressing mesenchymal stromal cells to ischemic brain enhances post-stroke recovery partially through PRDX4-mediated blood-brain barrier preservation. *Theranostics* **8**, 5929-5944 (2018).

3 Aasen, T. & Izpisua Belmonte, J. C. Isolation and cultivation of human keratinocytes from skin or plucked hair for the generation of induced pluripotent stem cells. *Nat Protoc* **5**, 371-382 (2010).

4 Liu, J. *et al.* Islet-1 overexpression in human mesenchymal stem cells promotes vascularization through monocyte chemoattractant protein-3. *Stem Cells* **32**, 1843-1854 (2014).
5 Zhang, X. *et al.* CXCR5-Overexpressing Mesenchymal Stromal Cells Exhibit Enhanced Homing and Can Decrease Contact Hypersensitivity. *Mol Ther* **25**, 1434-1447 (2017).

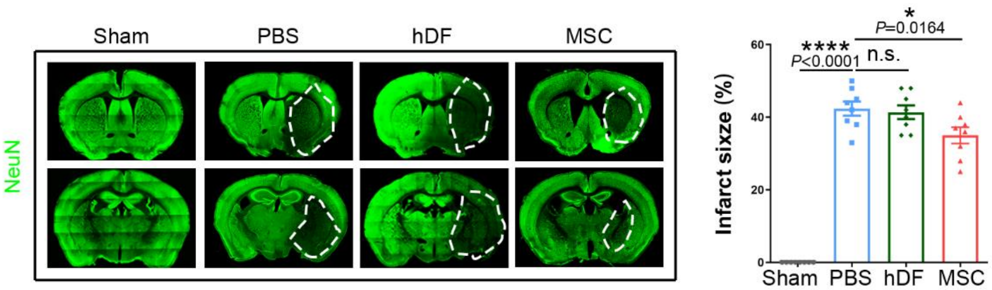

Supplementary Figure 1. MSC administration reduced infarct size.

Representative NeuN-stained coronal sections in different groups. Infarct volumes of different groups were determined at 7days post-stroke and quantified on NeuN (green)-stained coronal cerebral sections (n=8; one-way ANOVA).The quantified results are presented as the median ± min to max value.

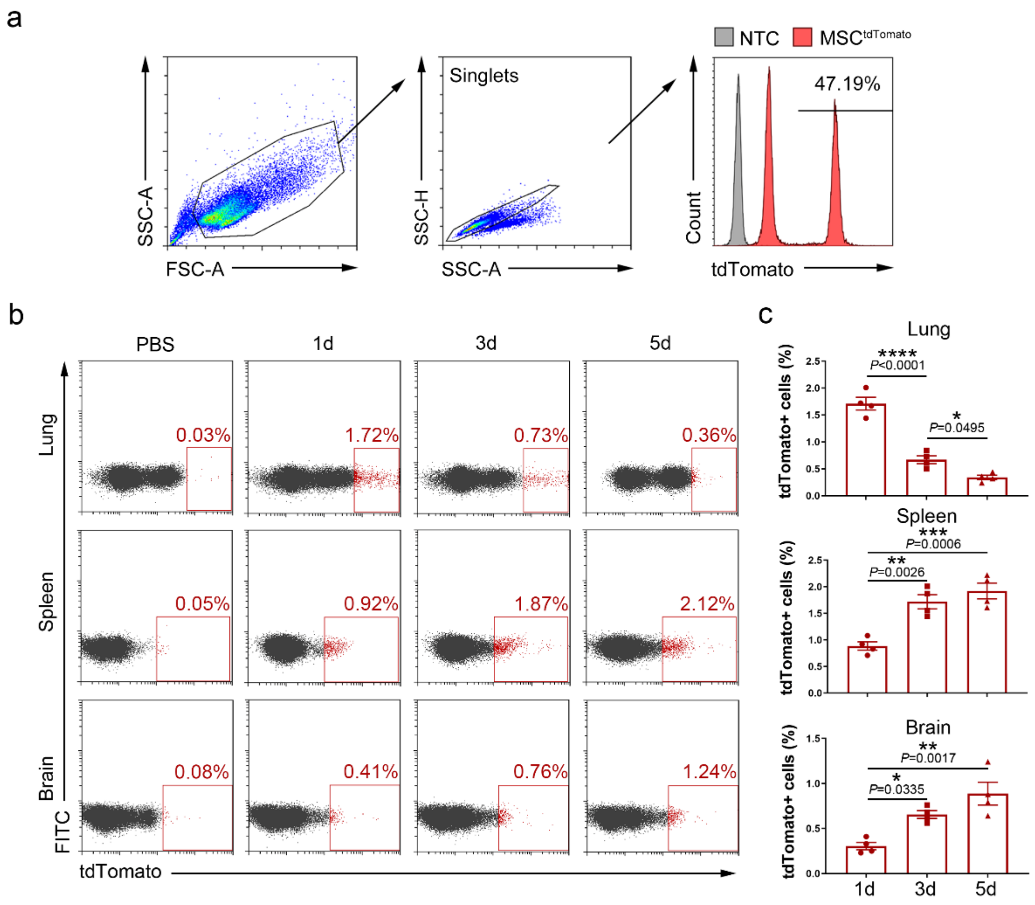

Supplementary Figure 2. Infused MSCs preferentially migrate to the spleen.

(a) MSCs expressing tdTomato were isolated using FACS. (b) Representative flow cytometric dot plots for tdTomato labeling of cells from freshly isolated lung, spleen, and brain of transplanted mice. (c) Quantitative analyses of percentage of $tdTomato^+$ cells in the lung, spleen, and brain of MSC- or PBS-treated mice at the indicated time points after cell transplantation. n=4.

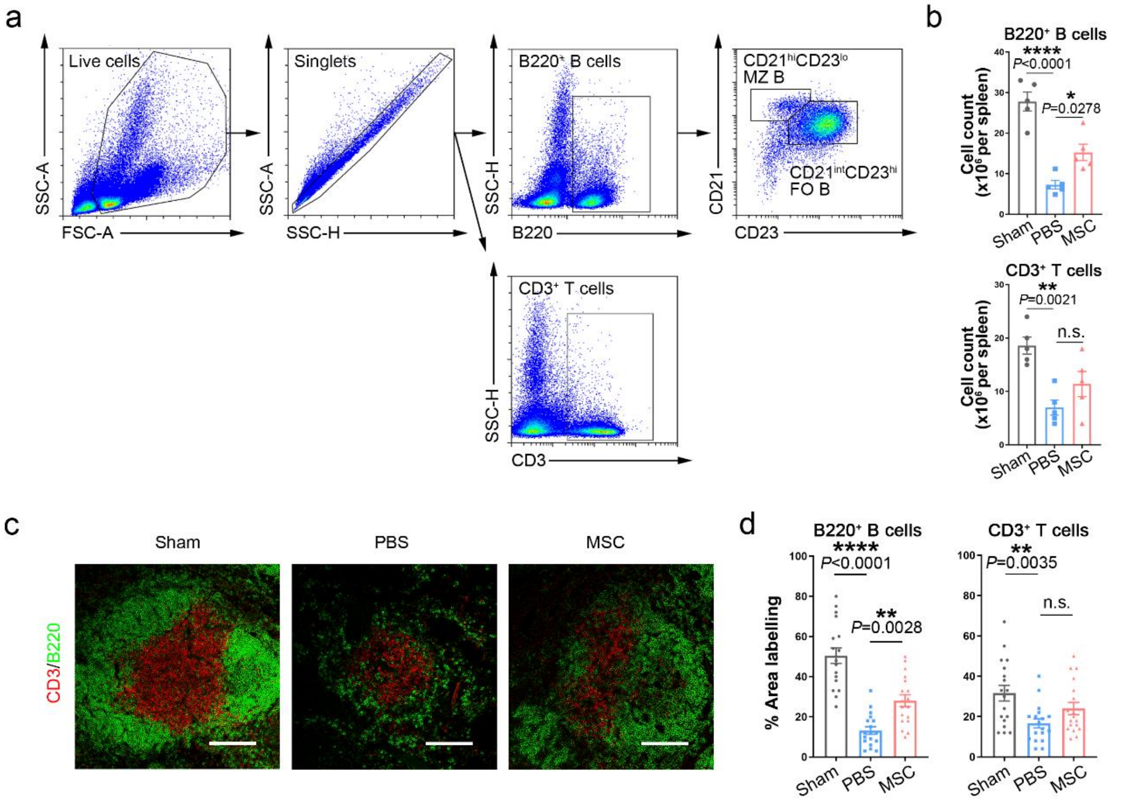

Supplementary Figure 3. MSC therapy prevents loss of splenic B cells.

C57/BL6 mice were subjected to 40 min of MCAO followed 6 h later by PBS or MSC treatment. Animals were sacrificed at 5 days post-stroke for FACS and immunofluorescence analyses. (a) Gating strategy for $CD3^+$ T cells, $B220^+$ B cells, $B220^+CD21^{hi}CD23^{lo}$ MZB cells, $CD21^{int}CD23^{hi}$ and FOB cells. (b) MSC administration increased B cell counts at 5days after MCAO, while had no significant impact on T cells. (c) Immunofluorescent labeling of splenic $B220^+$ B cells and $CD3^+$ T cells in sham operated or MCAO mice. Scale bar: 100 µm. (d) Quantification of $B220^+$ B cells (green) and $CD3^+$ T cells (red) labeling demonstrates the increase in the B cell area (but not the T cell area) of MSC-treated mice compared to the PBS group. n=3 mice per group. Six independent fields of view per mouse.

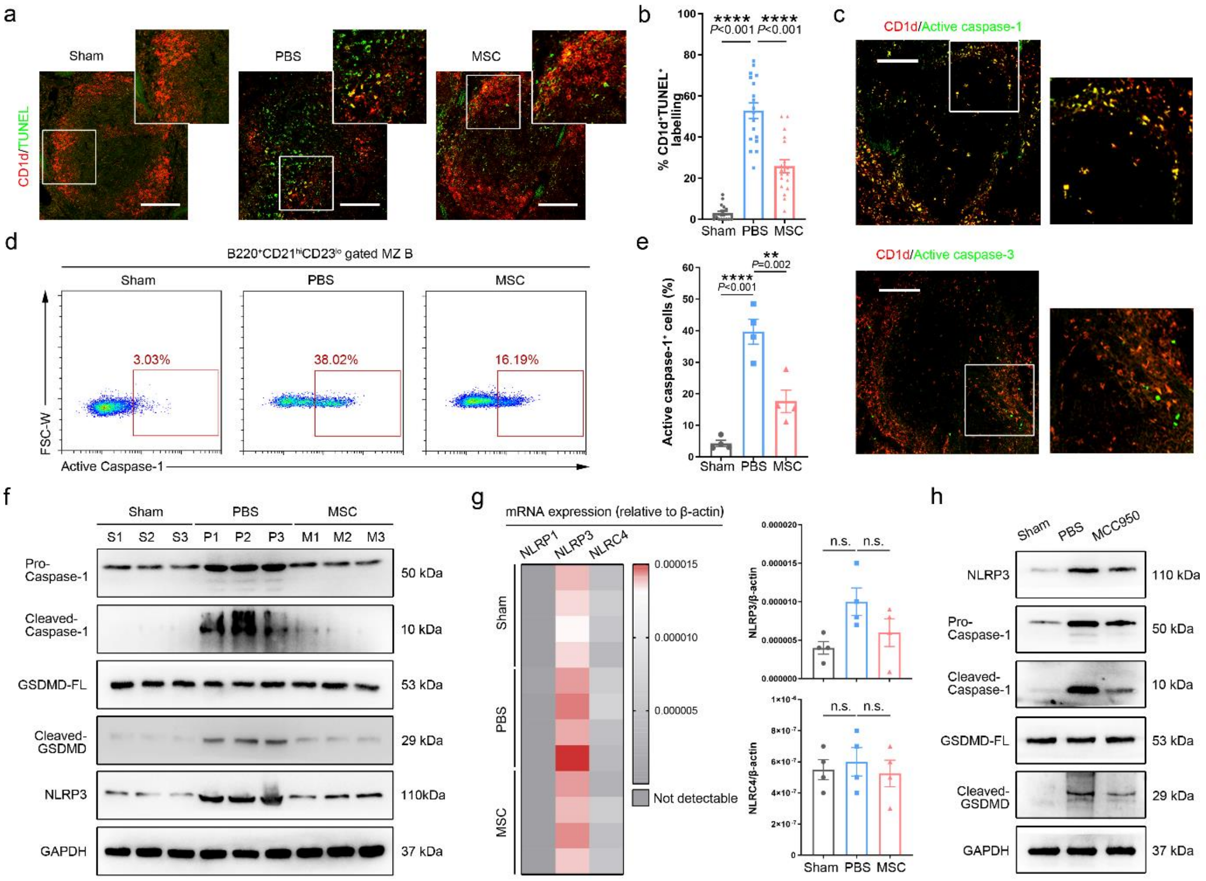

Supplementary Figure 4. MSCs attenuate caspase1-dependent MZ B cell death, potentially through the inhibition of NLRP3 activation.

(a) Representative images demonstrated TUNEL (green) co-labelled with CD1d (red) in spleen at 5days post-MCAO. Scale bar: 100μm. (b) Quantification of the percentage of TUNEL^{+} cells that colocalized with CD1d. n=3 mice per group. Six independent fields of view per mouse. (c) Immunofluorescence of CD1d (red) and active caspase-1 (upper panel) or caspase-3 (lower panel) 5 days after MCAO. CD1d labeling mainly co-localizes with active caspase-1, but not active caspase-3. Scale bar: 100 μm. (d-e) MSC treatment reduces the caspase-1 activation in B220^{+}CD21^{hi}CD23^{lo} MZ B cells induced by MCAO at 5 days after stroke. n=4. (f) B220^{+}CD21^{hi}CD23^{lo} MZB cells were isolated from sham operated and MCAO mice with or without MSC treatment at 5 days after stroke. Cells were lysed and the expression levels of pro-caspase-1, cleaved caspase-1, full-length gasdermin D (GSDMD), and cleaved GSDMD were analyzed by Western blotting. (g) Heat map showing mRNA expression levels of the NLRP1, NLRP3, and NLRC4 inflammasomes measured using qRT-PCR at 5 days after stroke. n=4. (h)

Representative immunoblotting shows the NLRP3, cleaved caspase-1 and cleaved GSDMD in MZ B cell lysates from PBS- (negative control) and MCC950-treated mice.

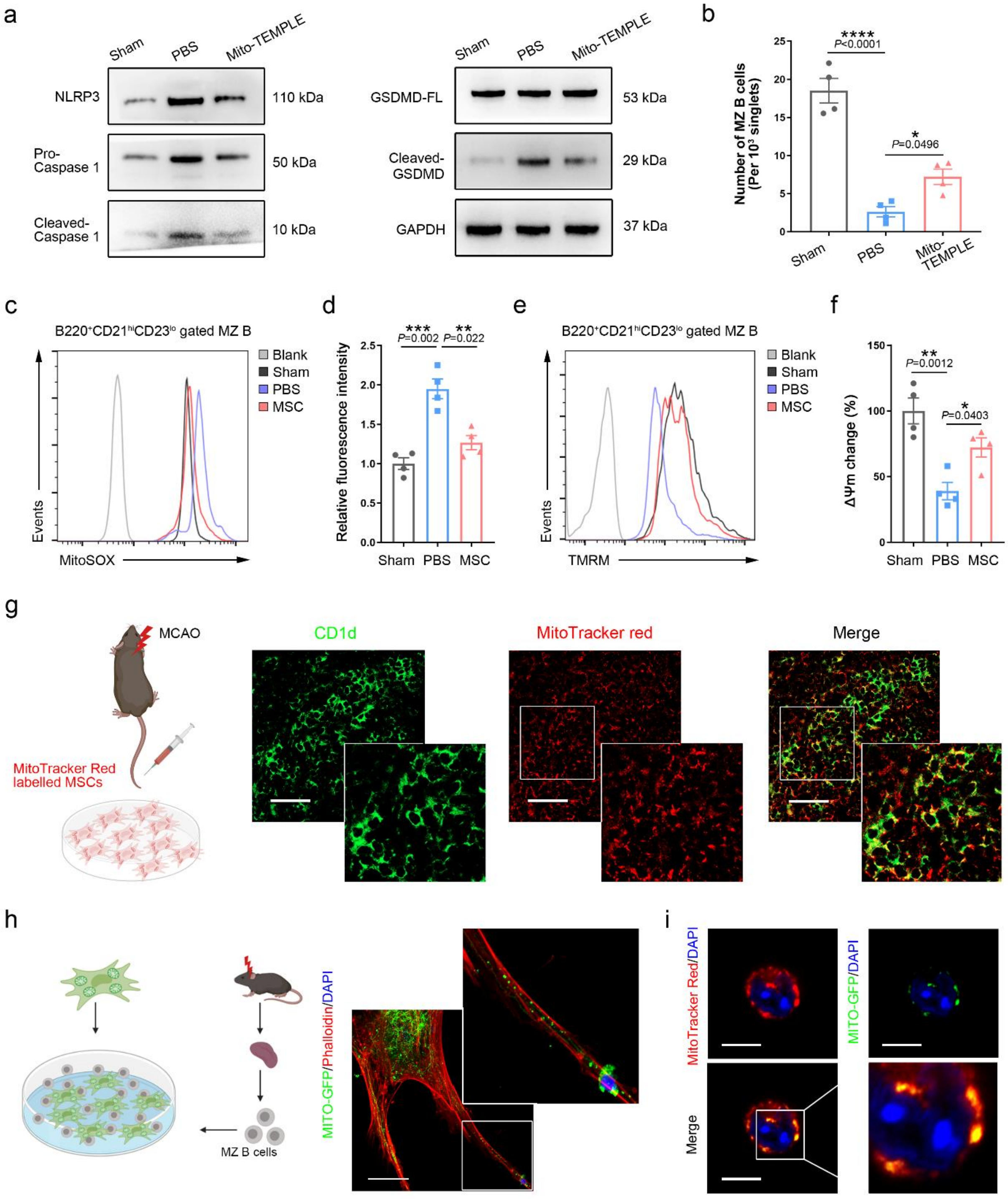

Supplementary Figure 5. MSCs transfer mitochondria to MZ B cells and maintain mitochondrial homeostasis in MZ B cells.

(a) C57BL/6 mice were subjected to 40 min MCAO and treated with mito-TEMPLE (i.p., 1 mg/kg/d) for 5 days. $B220^{+}CD21^{hi}CD23^{lo}$ MZB cells were isolated, lysed, and subjected to immunoblotting to determine the levels of cleaved caspase-1 and cleaved GSDMD. (b) The frequency of $B220^{+}CD21^{hi}CD23^{lo}$ MZB cells was measured by FACS at 5 days after the application of mito-TEMPLE. n=4. (c-d) Density plot analyses (c) and statistical analyses (d) of mitochondrial ROS levels in MZ B cells from sham operated and MCAO mice with or without MSC treatment, as measured by FACS. n=4. (e-f) Comparison of MMP using TMRM staining in $B220^{+}CD21^{hi}CD23^{lo}$ gated MZ B cells from sham operated and MCAO mice with or without MSC treatment, as assessed by FACS. n=4. (g) MSCs labeled with mito-tracker red were injected to C57BL/6 mice at 6 h after MCAO. Representative spleen images show mito-tracker-labeled MSC mitochondria (red) distributed in $CD1d^{+}$ (green) MZ B cells. Scale bar: 100 μm. (h) Left panel: Illustration of the system for co-culture of MSCs and MZ B cells isolated from different groups of mice. Right panel: Representative confocal image showing microtubule-mediated (red) mitochondria transfer (green) from MSCs to MZ B cells. Scale bar: 20 μm. (i) Representative image showing that mitochondria transferred from MSCs (MITO-GFP) were fused with mitochondria in MZB cells (labeled by mito-tracker red). Scale bar: 5 μm.
